# Quantifying Spatial Distribution of Ventilation Defects in Multiple Pulmonary Diseases With Hyperpolarized ^129^Xenon MRI

**DOI:** 10.1002/jmri.29627

**Published:** 2024-10-22

**Authors:** Abdullah S. Bdaiwi, Matthew M. Willmering, Jason C. Woods, Laura L. Walkup, Zackary I. Cleveland

**Affiliations:** ^1^ Center for Pulmonary Imaging Research, Division of Pulmonary Medicine Cincinnati Children's Hospital Medical Center Cincinnati Ohio USA; ^2^ Department of Pediatrics University of Cincinnati Cincinnati Ohio USA; ^3^ Imaging Research Center, Department of Radiology Cincinnati Children's Hospital Medical Center Cincinnati Ohio USA; ^4^ Department of Biomedical Engineering University of Cincinnati Cincinnati Ohio USA

**Keywords:** spatial distribution, ^129^Xe ventilation defects, hyperpolarized ^129^Xe, ventilation analysis

## Abstract

**Background:**

Hyperpolarized ^129^Xe MRI assesses lung ventilation, often using the ventilation defect percentage (VDP). Unlike VDP, defect distribution index (DDI) quantifies spatial clustering of defects.

**Purpose:**

To quantify spatial distribution of ^129^Xe ventilation defects using DDI across pulmonary diseases.

**Study Type:**

Retrospective.

**Subjects:**

Four hundred twenty‐one subjects (age = 23.1 ± 17.1, female = 230), comprising healthy controls (N = 60) and subjects with obstructive conditions (asthma [N = 25], bronchiolitis obliterans syndrome [BOS, N = 18], cystic fibrosis [CF, N = 90], lymphangioleiomyomatosis [LAM, N = 50]), restrictive conditions (bleomycin‐treated cancer survivors [BLEO, N = 14]; fibrotic lung diseases [FLD, N = 92]), bone marrow transplantation (BMT, N = 53), and bronchopulmonary dysplasia (BPD, N = 19).

**Field Strength/Sequence:**

3 T, two‐dimensional multi‐slice gradient echo.

**Assessment:**

Whole‐lung mean DDI was extracted from DDI maps; correlated with VDP (percent of pixels <60% of whole‐lung mean signal intensity) and pulmonary function tests (PFTs) including FEV_1_, FVC, and FEV_1_/FVC. DDI and DDI/VDP, a marker of defect clustering, were compared across diseases.

**Statistical Tests:**

Pearson correlation analysis and Kruskal–Wallis tests. *P* < 0.0056 for disease groups, *P* < 0.0125 for categories.

**Results:**

DDI was significantly elevated in BMT (8.3 ± 11.5), BOS (30.1 ± 57.5), BPD (16.0 ± 46.8), CF (15.4 ± 27.2), and LAM (12.6 ± 34.2) compared to controls (1.8 ± 3.1). DDI correlated significantly with VDP in all groups (*r* ≥ 0.56) except BLEO, and with PFTs in CF, FLD, and LAM (*r* ≥ 0.56). Obstructive groups had significantly higher mean DDI (14.0 ± 32.0) than controls (1.8 ± 3.0) and restrictive groups (4.0 ± 12.0). DDI/VDP was significantly lower in the restrictive group (0.6 ± 0.6) than controls (0.8 ± 0.6) and obstructive group (1.0 ± 1.0).

**Data Conclusion:**

DDI may provide insights into the distribution of ventilation defects across diseases.

**Evidence Level:**

3

**Technical Efficacy:**

Stage 2

## Introduction

Regional ventilation abnormalities can be assessed non‐invasively and with high sensitivity using hyperpolarized (HP) ^129^Xe MRI in a range of lung diseases.^1^ This typically involves inhaling a volume of HP ^129^Xe gas and imaging the distribution of the inhaled gas during a brief breath‐hold.^2^, ^3^ Within the resulting ventilation maps, regions displaying high HP ^129^Xe signal intensity represent hyperventilated lung volume, while regions that display abnormally low signal intensity represent obstructed airflow and thus reduced ventilation. While multiple methods have been proposed to quantify the extent of low signal intensity regions (ventilation defects), including linear binning^4^, ^5^ and K‐means clustering,^6^ the most commonly used metric is the percent of lung volume with signal intensity below a specified threshold (eg, 60% of whole‐lung mean).^2^, ^7^ This ventilation defect percentage (VDP), has become a valuable tool for evaluating ventilation abnormalities in a range of lung diseases, including asthma,^8^, ^9^ lymphangioleiomyomatosis (LAM),^10^ chronic obstructive pulmonary disease,^11^, ^12^ and cystic fibrosis (CF).^2^, ^7^

Although ^129^Xe VDP is an established and increasingly ubiquitous method to identify, evaluate and monitor lung diseases, it does not assess regional lung dysfunction. VDP also does not reflect the intrinsic spatial heterogeneity of lung pathophysiology, particularly defect clustering, which is necessary to understand regional severity and assess the role of airway size (i.e., generation) on impaired ventilation.^13^ Hence, a given numerical value of VDP (eg, 25%) could result from: 1) a small number of obstructions in airways generations 2–16 (segmental bronchi [generations 2–3]), subsegmental bronchi [4‐6] and bronchioles [7‐16], which yield large, segment‐level defects; 2) a large number of obstructions in the distal airways, which yield multiple, highly distributed defects; or 3) a mixture of proximal and distal obstructions. Thus, understanding the regional ventilation defect volume distribution is required to more fully characterize disease pathophysiology, disease progression, and therapy response.

To gain a deeper understanding of disease phenotypes, pathophysiology progression, and treatment efficacy, a method to quantify the spatial distribution of ventilation defects is required. To this end, the number of defects and largest contiguous defect have been considered along with VDP using ^3^He MRI in CF.^14^ More recently, Valk et al^15^ introduced a metric to assess the 2D spatial distribution of ventilation lung defects called the defect distribution index (DDI). This metric was applied to individuals with CF using proton lung imaging combined with matrix pencil decomposition.^16^ DDI quantifies the spatial “clustering” of defects, with low DDI reflecting diffuse obstruction and high DDI reflecting focal obstruction. It is an observer‐independent assessment of ventilation obstruction, suggesting it may be well‐suited for longitudinal analysis and interindividual comparisons—even in the context of multi‐site clinical studies.

Thus, the aim of this study was to extend the previously established 2D DDI technique^15^ to assess the 3D spatial distribution of ventilation using ^129^Xe MRI in a large dataset, including healthy subjects and those with both restrictive and obstructive pulmonary diseases.

## Materials and Methods

### Study Population

This study includes both retrospective and prospectively collected data. The retrospective data, consisting of 343 cases, were originally collected at Cincinnati Children's Hospital Medical Center and have been previously published.^4^, ^10^, ^17^, ^18^, ^19^, ^20^, ^21^, ^22^ The prospective data were collected in new studies, involving 78 participants, and are registered under U.S. Food and Drug Administration (IND‐123,577).

All studies were approved by the Cincinnati Children's Institutional Review Board. Written informed consent was obtained from all adult participants or parents of pediatric subjects, and age‐appropriate assent was obtained from pediatric participants only. This current study adheres to the ethical guidelines set forth in the original approvals and incorporates both the previously published retrospective data and the new prospective data.

From December 2014 and August 2023, ^129^Xe ventilation MRI datasets were collected from subjects who underwent imaging at Cincinnati Children's Hospital Medical Center for various purposes, including diagnosis, monitoring, and evaluation of treatment response for various pulmonary conditions.

Inclusion criteria included age >5 years, and ability to complete a 16‐second breath‐hold, with a variable success rate up to 71% in children.^20^ Exclusion criteria included active respiratory infection, chest tightness, or sinus infection within 1 week of MRI, baseline pulse oximetry (SpO2) ≤95%, pregnancy or positive urine pregnancy test (females of reproductive age), and standard contraindication of MRI. Pulmonary function tests (PFTs), including forced expiratory volume in 1 second (FEV_1_), forced vital capacity (FVC), and FEV_1_/FVC, were also retrieved from patients' medical records, contingent on their availability. In subjects with multiple exams during the study period, only one study (earliest study) was included in the analysis.

### ^129^Xe Polarization and Delivery

Isotopically enriched xenon (85% ^129^Xe; gas mixture: 1 or 2% Xe, 10% N_2_, 88 or 89% He; Linde Elec & Specialty Gasses Inc., Alpha, NJ, USA) was polarized to 15%–35%^23^ (Models 9810 or 9820A, Polarean Imaging, plc, Durham, NC, USA) and dispensed into Tedlar bags (Jensen Inert Products, Coral Springs FL, USA). Total xenon dose was 1 L for adults or 1/6th of total lung capacity estimated according to American Thoracic Society guidelines (maximum of 1 L) for pediatric subjects.^24^ After inhalation, ^129^Xe ventilation images were acquired during a ≤16 second breath‐hold in the presence of a medical professional, who monitored heart rate and blood oxygenation throughout the protocol.

### MRI Data Acquisition

HP ^129^Xe ventilation images were acquired using 3 T Philips Achieva or Ingenia scanners (Philips Healthcare, Best, the Netherlands) and either a home‐built dual‐loop, single channel ^129^Xe transmit‐receive coil or a flexible transmit/receive ^129^Xe chest coil (Clinical MR Solutions, Brookfield, WI, USA). Multi‐slice (2D) gradient‐recalled echo (GRE) or spiral sequences were used. Images were acquired in axial or coronal slice orientation (Table 1). Image acquisition parameters included: TE/TR = 3.75/7.73 msec for GRE and 1.52/12.6 msec for spiral, flip angle = 10–12° for GRE and 10–30° for spiral (depending on number of excitations^25^), field of view = 275–325 mm^2^, in‐plane resolution = 3 × 3 mm^2^, slice thickness = 15 mm, slice gap = 0–10 mm, number of slices = 10–20, bandwidth (Hz/pixel) = 270 for GRE and 160 for spiral. All images were reconstructed on‐line using the standard vendor reconstruction pipeline.

**TABLE 1:** Subjects' Demographics and MRI Details

Disease Category	Disease Groups	N	Age (Year)^a^	Sex		Slice Orientation		Sampling Type		Published Data
				F	M	Axial	Coronal	Cartesian	Spiral	N (Ref.)
Healthy	Control	60	15.4 ± 7.6 (5.4–40.3)	23	37	47	13	47	13	23, 51, 63 (4, 18, 22)
Obstructive	Asthma	25	13.3 ± 8.3 (7.3–50.6)	13	12	25	‐	25	‐	22, 28, 21 (4, 17, 18)
	BOS	18	14.9 ± 5.9 (5.1–25.2)	7	11	18	‐	18	‐	14 (18)
	CF	90	15.7 ± 7.5 (5.5–46.0)	44	46	57	33	82	8	37, 101, 37 (4, 18, 21)
	LAM	50	47.3 ± 10.6 (20.0–71.0)	49	1	50	‐	50	‐	29, 22, 61 (4, 10, 18)
Restrictive	BLEO	14	31.0 ± 8.0 (19.5–44.1)	3	11	‐	14	‐	14	‐
	FLD	92	35.2 ± 21.5 (4.5–81.0)	54	38	37	55	37	55	7, 32 (4, 18)
Others	BMT	53	11.7 ± 4.6 (5.0–26.0)	28	25	53	‐	53	‐	47, 81, 23 (4, 18, 19)
	BPD	19	12.5 ± 8.5 (5.0–36.2)	10	9	19	‐	19	‐	20, 24 (18, 20)
Total		421	23.1 ± 17.1 (4.5–81.0)	231	190	306	115	331	90	

BLEO = childhood‐cancer survivors who received bleomycin; BMT = bone‐marrow transplantation; BOS = bronchiolitis‐obliterans syndrome; BPD = bronchopulmonary dysplasia; CF = cystic fibrosis; FLD = fibrotic lung diseases; LAM = lymphangioleiomyomatosis.

^a^ Values are mean ± SD (range).

For thoracic cavity segmentation, a ^1^H scan was acquired during a separate breath‐hold with a volume‐matched air bag. This was performed using the same sequence type and acquisition parameters as the ^129^Xe image except with the following parameters: TE/TR = 0.35/1.98 msec for GRE and 0.67/4.9 msec for spiral, flip angle = 5° for GRE and 8° for spiral, bandwidth = 100 Hz/pixel for GRE and 300 Hz/pixel for spiral.

### VDP and DDI Quantification

Binary masks of lung parenchyma (excluding large airways) were generated manually using ^1^H images as a reference guide. The analyses were conducted by an analyst with over 2 years of experience. All generated masks were subsequently reviewed and edited by all co‐authors of this manuscript to ensure accuracy and reliability. To mitigate signal variation due to B_1_‐field inhomogeneity, images were corrected using N4ITK bias‐field.^26^

**FIGURE 1:**
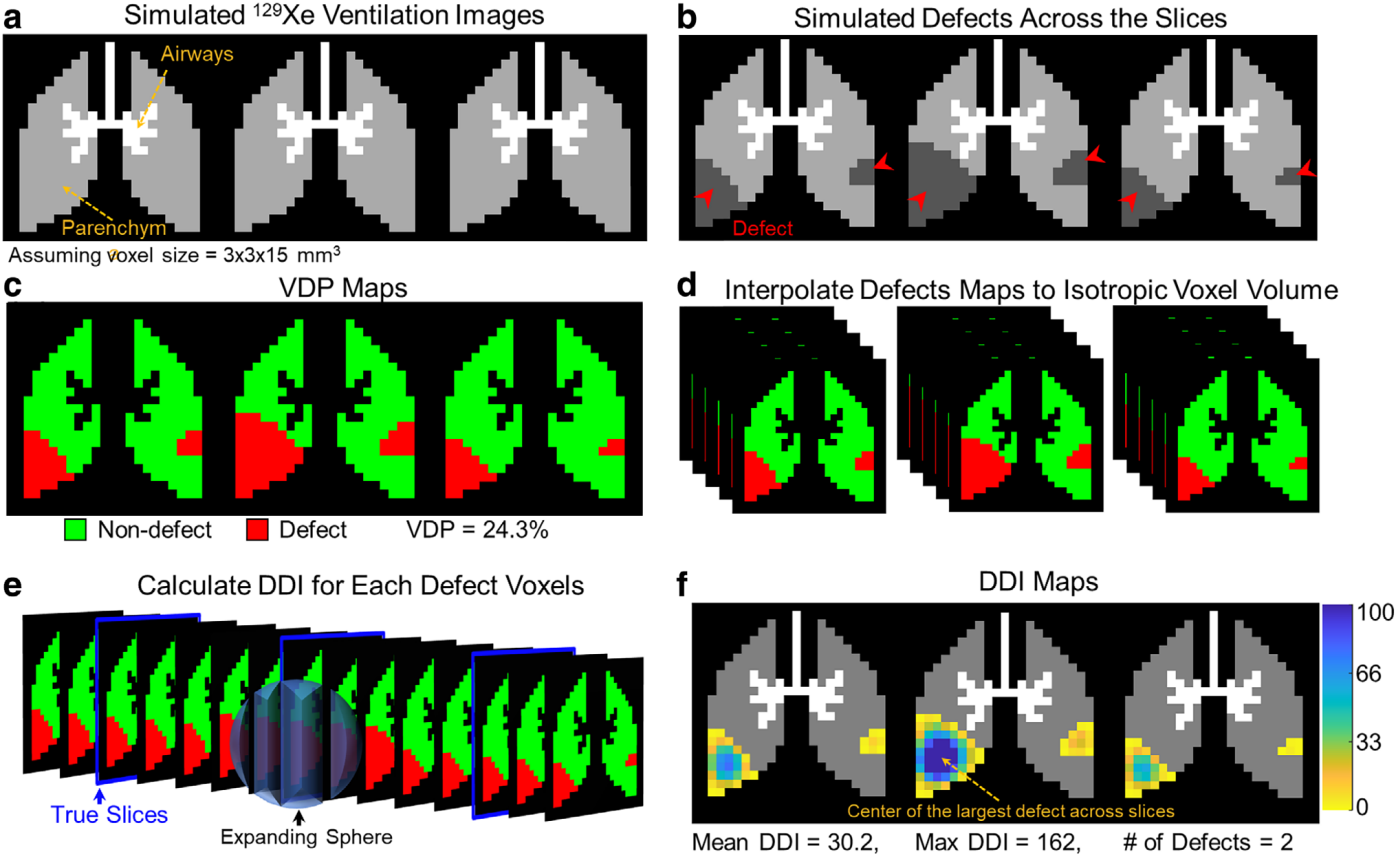
3D defect distribution index (DDI) workflow. (**a**) Simulated ^129^Xe ventilation images. (**b**) Simulated defects across slices. (**c**) Ventilation defect percentage (VDP) is computed. (**d**) Defect maps interpolated into 3D with an isotropic voxel volume based on the slice thickness. (**e**) For each defect voxel in the true slices, DDI is computed by centering a sphere around each defect voxel and gradually increasing its radius until the fraction of defect voxels falls below 50% of the total voxels inside the sphere. (**f**) The DDI output map is generated according to Eq. A9 and shows the DDI values for all defect voxels (see Appendix A in the Supplemental Material).

VDP and DDI were computed as depicted for the simulated ventilation images in Fig. 1. Defect regions in the ^129^Xe ventilation images (red) were first identified as voxels with signal intensity <60% of the whole‐lung mean^2^, ^4^, ^10^ and VDP calculated as the number of defect voxels as a percentage of the total lung voxels (Fig. 1a–c). DDI was then calculated based on an expanded version of the DDI technique (Fig. 1d–f) introduced by Valk et al,^15^ which originally quantified defect distribution in 2D proton imaging by placing circles around defect voxels and expanding the radius until defect pixels fell below 50% of the total pixels within the circle. In this study, the method was adapted for 3D assessment by replacing 2D circles with spheres while retaining the 50% threshold.^27^ To address anisotropic voxel size (eg, 3 × 3 × 15 mm^3^) and slice gaps, slices were interpolated using nearest‐neighbor interpolation until isotropic voxel size (eg, 3 × 3 × 3 mm^3^) was achieved. For each defective voxel, a sphere was centered and its radius incrementally increased until the fraction of defect voxels within the sphere fell below 50%. The resulting sphere radius was then used to calculate a cluster score (Eq. A7 in Appendix A in the Supplemental Material), which determined the DDI value (Eq. A9 in Appendix A), as detailed in Appendix A in the Supplemental Material. All calculations were performed in MATLAB (Mathworks, Natick, MA, USA).

### Synthetic Lung Imaging

To demonstrate the sensitivity and scalability of the DDI, synthetic lung images were generated with a fixed VDP of 20% using MATLAB. These synthetic lungs exhibited different levels of defect clustering (low, medium, and high), as well as variations in the progression of defects, including both improvement (reduction in the size and/or number of defects, indicating better lung ventilation) and worsening (increase in the size and/or number of defects, indicating declining lung ventilation). Additionally, synthetic images (with low, medium, and high clustering) were generated with different lung sizes (5040, 12,200, 20,045 voxel^3^) to investigate the scalability of DDI.

### Statistical Analysis

The associations of mean DDI with VDP and PFTs were assessed using linear regression and Pearson's correlation coefficient (*r*) in all disease groups, where significance was defined as *P* < 0.05. To facilitate comparisons of DDI across all disease groups while accounting for differences in VDP, DDI was normalized by VDP (DDI/VDP). The Kruskal–Wallis test was performed to assess the significance of DDI and DDI/VDP differences between disease groups. To account for multiple comparisons, unadjusted *P*‐values from pairwise comparisons were compared to an adjusted significance threshold (Bonferroni correction) of *P* < 0.0056 (0.05/9) for identifying statistically significant differences in pairwise comparisons between disease groups. Differences in DDI and DDI/VDP were also evaluated between slice orientations (axial and coronal) using a two‐tailed *t*‐test, with significance level defined as *P* < 0.05 in healthy control, CF and fibrotic lung diseases (FLD) groups.

Additionally, the significance of DDI and DDI/VDP among the four disease categories (healthy control, obstructive, restrictive, and others) was determined using the Kruskal–Wallis test. In this case, the significance threshold was adjusted to *P* < 0.0125 (0.05/4) to determine statistically significant differences in pairwise comparisons between categories.

## Results

A total of 421 subjects (23.1 ± 17.1, 231 female) were included in the analysis and 60 were healthy controls. Three hundred sixty‐one patients were grouped according to their underlying pulmonary diseases: bleomycin‐treated childhood cancer survivors (N = 14), patients with asthma (N = 25), those with bronchiolitis obliterans syndrome (BOS, N = 18), bone marrow transplantation recipients (BMT, N = 53), patients with bronchopulmonary dysplasia (BPD, N = 19), patients with CF (N = 90), patients with fibrotic lung diseases (FLD, N = 92) encompassing idiopathic pulmonary fibrosis (IPF) and other fibrosing interstitial lung disease (ILD) and patients with lymphangioleiomyomatosis (LAM, N = 50). Patients were also re‐categorized to obstructive lung disease (asthma, BOS, CF, and LAM), restrictive lung disease groups (BLEO and FLD) and “others” (BMT and BPD). Subject demographics and brief description of MRI acquisition are provided in Table 1.

### Simulated Defects Distribution

**FIGURE 2:**
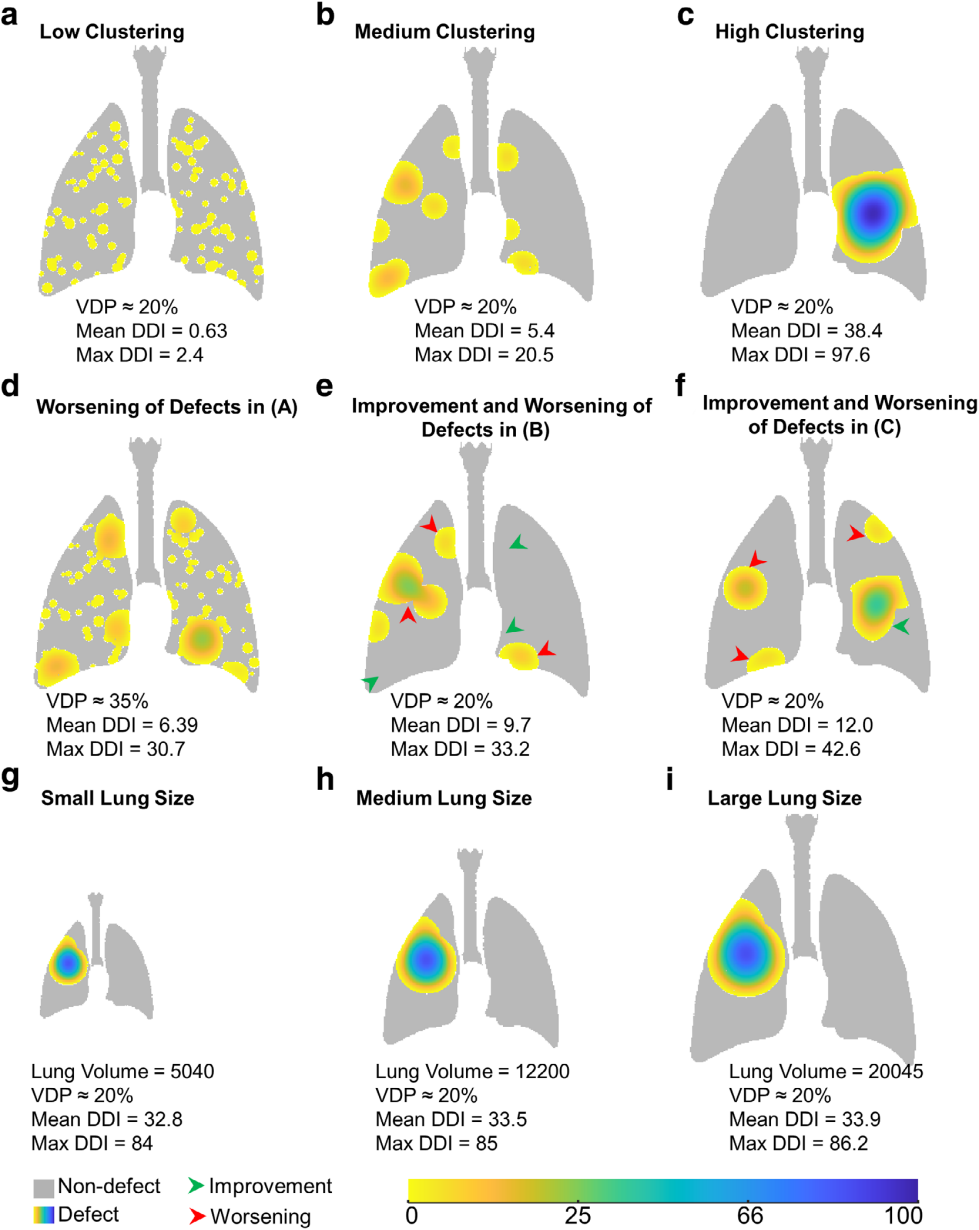
Illustration of DDI applied to artificial lung defect maps, showcasing varied defect distributions and sizes. In cases of (**a**) low clustering, (**b**) medium clustering, and (**c**) high clustering, simulated lungs maintain a fixed VDP of 20%. Despite uniform VDP, DDI varies depending on the specific defect distribution pattern. Case (**d**) demonstrates a simulated worsening of the defects in (a), with both VDP and DDI increasing. Conversely, case (**e**) shows simulated improvement and worsening of the defects in (b), with some defects disappearing while others enlarge. In this case, VDP remains unchanged, while DDI increases. Similarly, case (**f**) shows simulated improvement and worsening of the defects in (c), where large defects shrink, and new defects emerge. VDP remains constant, yet DDI has increased. Additionally, (**g–i**) highlight the scalability of DDI, as lung images with identical defect patterns (20% defect) but with differing numbers of lung voxels (i.e., sizes or resolutions) show unchanged DDI values.

In Fig. 2, different defect distribution patterns, characterized by varying levels of clustering (low, medium, and high), are depicted (a–c, respectively). All three patterns yielded an identical VDP of 20%. Despite this, the mean DDI differed, being 0.63, 5.4, and 38.4, for low, medium, and high clustering, respectively. In case (d), a deterioration of the simulated lung function in case (a) is shown. This deterioration led to an increase in both the VDP (to 35%) and DDI (to 6.39). Conversely, case (e) shows a combination of improving and worsening of defects in the simulated case (b). In this case, some defects vanished while others expanded in size. The resulting VDP was unchanged (20%), while the DDI increased (from 5.4 to 9.7). Similarly, case (f) shows an example based on (c) where large defects shrank, and new defects emerged. The VDP was again unchanged (20%) while the DDI decreased (from 38.4 to 12). Furthermore, cases (g–i) demonstrate the scalability of DDI with respect to lung volume. Even when lung images exhibit the same defect percentage (eg, 20% defect), the DDI values remain consistent regardless of the varying numbers of lung voxels (representing different sizes or resolutions).

### Correlations of DDI, VDP, and PFTs in Each Group

**FIGURE 3:**
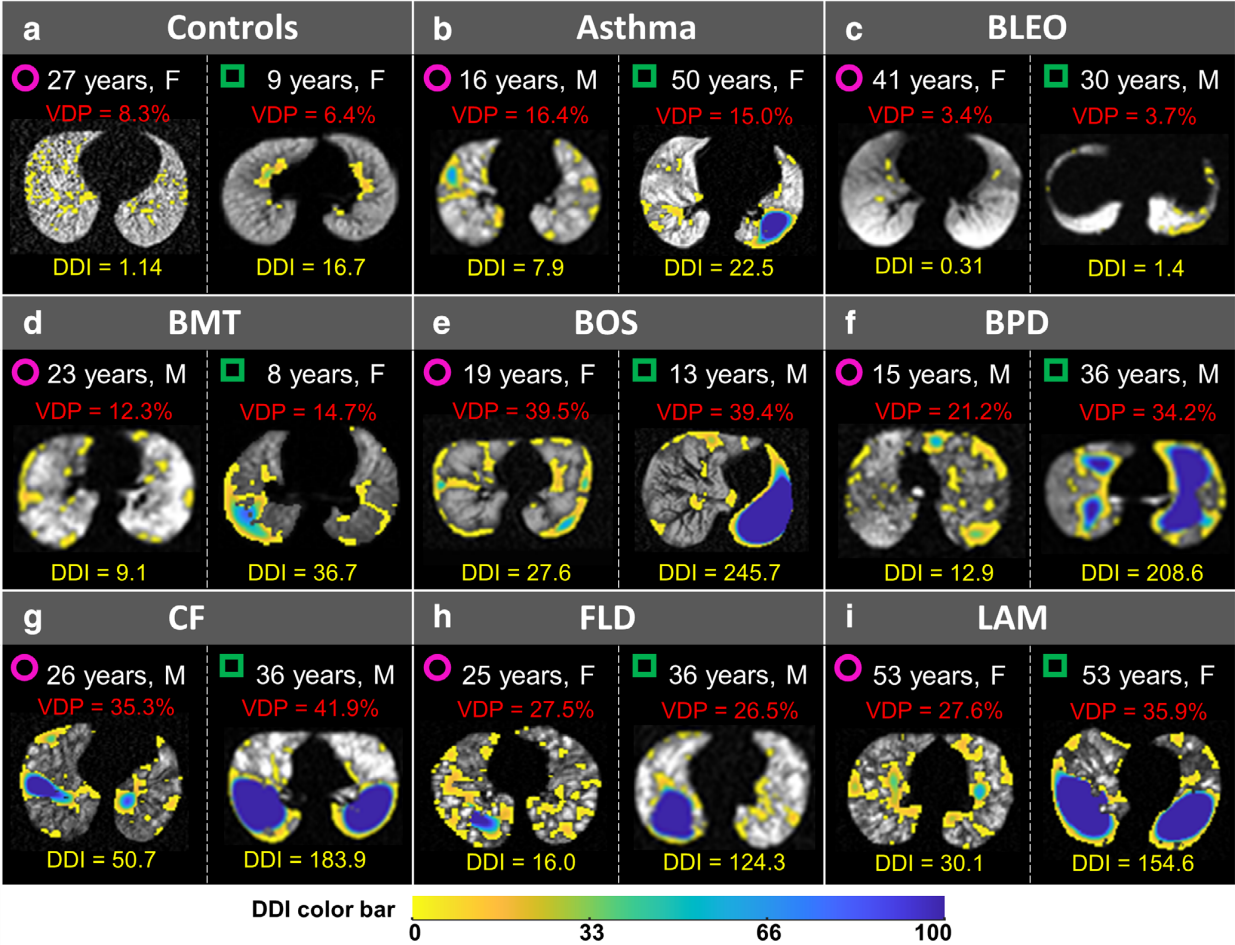
A representative slice from extreme cases across subject groups. Despite similar VDP values, the mean DDI maps show differences in the examples in each group.

As in the simulations, cases of obstructed ventilation were observed in vivo which result in nearly identical VDP but different DDI values. Representative slices showing discordant VDP and DDI were selected from each group—including healthy subjects—and are shown in Fig. 3. Notably, maps with a low mean DDI show scattered defects, while those with a high mean DDI show more clustered defects.

**FIGURE 4:**
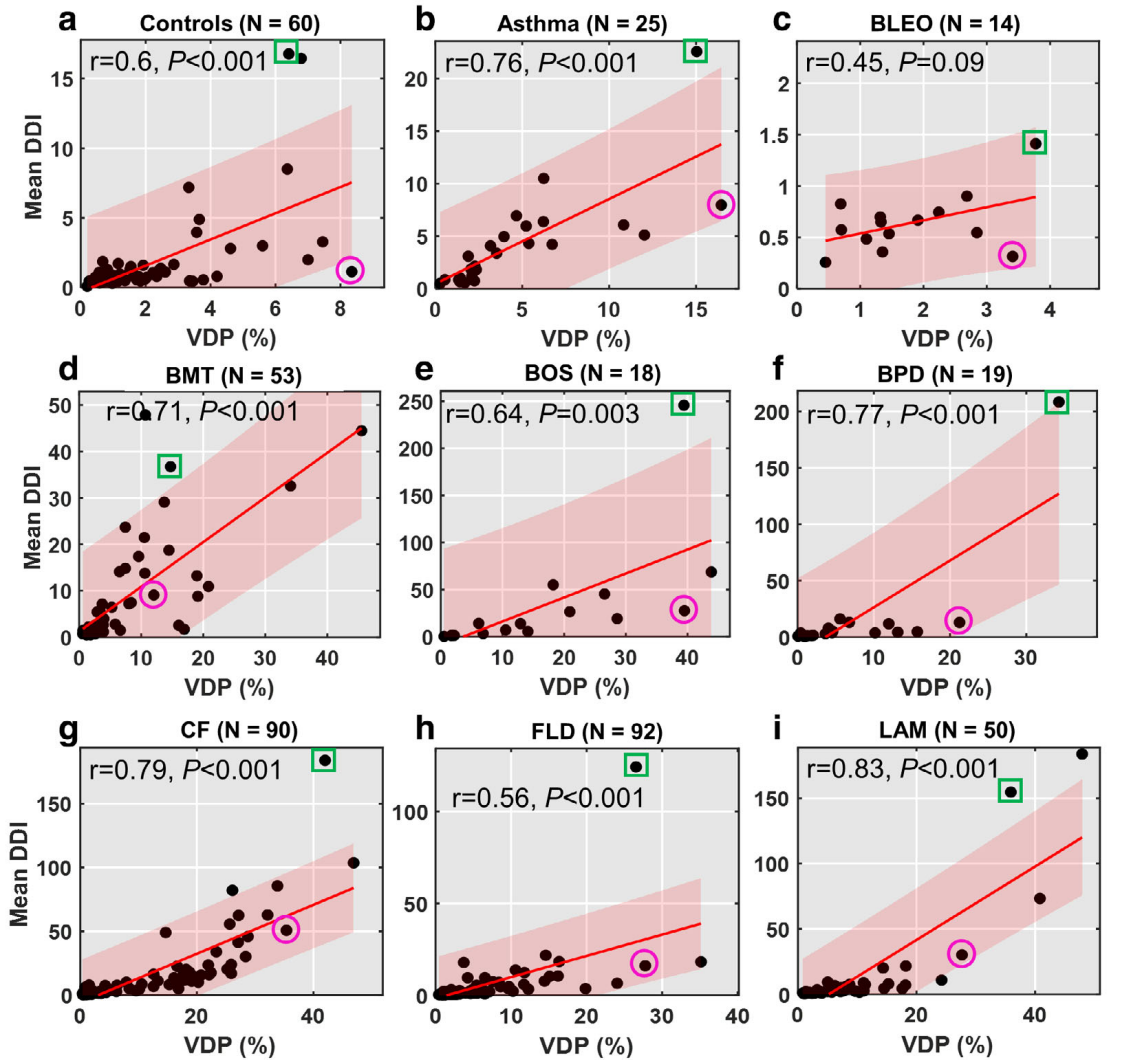
Scatter plots depicting correlations between DDI and VDP across all groups. For most groups, DDI correlates significantly with VDP (*r* ≥ 0.56), except for BLEO, where the correlation is not significant (*P* = 0.09). The 95% confidence interval is shaded, and the least squares fit is represented by the line. The extreme cases highlighted in Fig. 3 are denoted with green squares and magenta circles.

Table 2 provides a summary of the mean values for DDI, VDP, and PFTs across all groups. Table 3 and Figure 4 present the correlations between DDI, VDP, and PFTs for each group. Note, in Fig. 4, the representative cases shown in Fig. 3 are highlighted with green squares and magenta circles. DDI correlated significantly with VDP in all groups (*r* ≥ 0.56), except for BLEO (*P* = 0.09) which had the smallest number of subjects (N = 14). DDI did not correlate significantly with PFT measures (Figs. S1–S3 in the Supplemental Material) for the Control (*P* [FEV_1_] = 0.13, *P* [FVC] = 0.38, *P* [FEV_1_/FVC] = 0.21), Asthma (*P* [FEV_1_] = 0.2, *P* [FVC] = 0.4, *P* [FEV_1_/FVC] = 0.54), BLEO (*P* [FEV_1_] = 0.85, *P* [FVC] = 0.96, *P* [FEV_1_/FVC] = 0.64), BMT (*P* [FEV_1_] = 0.10, *P* [FVC] = 0.86), and BOS (*P* [FEV_1_] = 0.10, *P* [FVC] = 0.26, *P* [FEV_1_/FVC] = 0.06) groups. However, in the CF, FLD, and LAM groups, DDI correlated significantly with PFT measures. Additionally, VDP correlated significantly with PFTs (at least one test) in all groups (Table 2, Figs. S4–S6 in the Supplemental Material), except for Control group (*P* [FEV_1_] = 0.49, *P* [FVC] = 0.72, *P* [FEV_1_/FVC] = 0.52).

**TABLE 2:** Summary of Defect Distribution Index (DDI), Ventilation Defect Percentage (VDP), and Pulmonary Function Tests Across All Groups

Disease Category	Disease Groups	DDI	VDP	FEV_1_	FVC	FEV_1_/FVC
Healthy	Control	60 1.8 ± 3.1 (0.1–16.7)	60 2.27 ± 1.98 (0.20–8.34)	42 98.9 ± 15.2 (69–166)	42 102.5 ± 14.5 (66–156)	42 96.8 ± 8.9 (77–143)
Obstructive	Asthma	25 4.3 ± 4.6 (0.49–22.5)	25 4.7 ± 4.4 (0.27–16.4)	24 94.1 ± 17.9 (63–124)	24 106.7 ± 15.3 (68–131)	24 88.1 ± 10.4 (65–104)
	BOS	18 30.18 ± 57.5 (0.33–245)	18 15.4 ± 14.6 (0.53–43.8)	10 67.8 ± 27.0 (29–105)	10 81.9 ± 16.9 (54–108)	10 79.9 ± 20.4 (50–107)
	CF	90 15.4 ± 27.2 (0.28–183)	90 11.1 ± 11.3 (0.41–46.8)	87 93.2 ± 19.2 (38–131)	87 99.7 ± 15.5 (58–133)	87 93.1 ± 11.2 (53–111)
	LAM	50 12.6 ± 34.2 (0.38–183)	50 9.7 ± 10.1 (0.8–47.9)	37 88.5 ± 22.8 (35–132)	37 97.2 ± 17.1 (59–135)	37 91.4 ± 18.5 (36–120)
Restrictive	BLEO	14 0.64 ± 0.29 (0.25–1.4)	14 1.8 ± 1.0 (0.45–3.7)	12 96.8 ± 13.7 (73–129)	12 100.8 ± 15.9 (75–131)	12 96 ± 6.2 (83.9–121)
	FLD	92 4.6 ± 13.4 (0.30–124)	92 5.4 ± 6.5 (0.43–35)	51 93.1 ± 19.3 (41–125)	51 93.2 ± 19.1 (39–125)	51 100.3 ± 11 (64.7–130)
Others	BMT	53 8.3 ± 11.5 (0.49–47.8)	64 7.3 ± 8.5 (0.57–45.5)	42 79.3 ± 22 (30–121)	42 84.5 ± 20.1 (31–130)	42 94.2 ± 14.6 (40–110)
	BPD	19 16.0 ± 46.8 (0.51–208)	19 7.5 ± 8.7 (0.05–34.2)	11 66 ± 19 (40–104)	11 90 ± 15 (68–123)	11 72 ± 21 (40–103)
*P*‐values*		**<0.001**	**<0.001**	**<0.001**	**<0.001**	**<0.001**
*P*‐values†		**<0.001**	**<0.001**	**<0.001**	**<0.001**	**<0.001**

*Note*: Bold values indicate statistical significance (*p* < 0.05)

Data are presented as sample size, mean ± SD (range).

DDI = Defect Distribution Index; VDP = Ventilation Defect Percentage; FEV_1_ = Forced Expiratory Volume in 1 second; FVC = Forced Vital Capacity; BLEO = childhood‐cancer survivors who received bleomycin; BMT = bone‐marrow transplantation; BOS = bronchiolitis‐obliterans syndrome; BPD = bronchopulmonary dysplasia; CF = cystic fibrosis; FLD = Fibrotic lung diseases; LAM = lymphangioleiomyomatosis.

* *P* < 0.05 indicates statistical significance among the disease category.

† *P* < 0.05 indicates statistical significance among the disease groups.

**TABLE 3:** Correlation Coefficients Between DDI, VDP, and PFTs Across All Groups

Disease Category	Disease Groups	DDI vs. VDP	DDI vs. FEV_1_	DDI vs. FVC	DDI vs. FEV_1_/FVC	VDP vs. FEV_1_	VDP vs. FVC	VDP vs. FEV_1_/FVC
Healthy	Control	0.6 (**<0.001**)	−0.23 (0.13)	−0.13 (0.38)	−0.19 (0.21)	−0.1 (0.49)	−0.05 (0.72)	−0.1 (0.52)
Obstructive	Asthma	0.76 (**<0.001**)	−0.27 (0.20)	−0.42 (0.40)	0.13 (0.54)	−0.41 (**0.04**)	−0.46 (**0.02**)	−0.08 (0.7)
	BOS	0.64 (**0.003**)	−0.53 (0.10)	−0.39 (0.26)	−0.60 (0.06)	−0.76 (**0.009**)	−0.6 (0.06)	−0.82 (**0.003**)
	CF	0.79 (**<0.001**)	−0.57 (**<0.001**)	−0.36 (**<0.001**)	−0.62 (**<0.001**)	−0.63 (**<0.001**)	−0.43 (**<0.001**)	−0.62 (**<0.001**)
	LAM	0.83 **(<0.001)**	−0.50 (**0.001**)	0.23 (0.16)	−0.72 (**<0.001**)	−0.55 (**<0.001**)	0.03 (0.83)	−0.66 (**<0.001**)
Restrictive	BLEO	0.45 (0.09)	−0.05 (0.85)	−0.01 (0.96)	−0.14 (0.64)	0.17 (0.57)	0.49 (0.1)	−0.72 (**0.007**)
	FLD	0.56 (**<0.001**)	−0.30 (**0.03**)	−0.32 (**0.001**)	−0.45 (**<0.001**)	−0.38 (**0.004**)	−0.29 (**0.03**)	−0.13 (0.32)
Others	BMT	0.71 (**<0.001**)	−0.25 (0.10)	0.02 (0.86)	−0.47 (**0.001**)	−0.43 (**0.003**)	−0.003 (0.72)	−0.73 (**<0.001**)
	BPD	0.77 (**<0.001**)	−0.2 (0.3)	0.07 (0.8)	−0.4 (0.1)	−0.5 (0.1)	0.05 (0.85)	−0.62 (**0.03**)

*Note*: Bold values indicate statistical significance (*p* < 0.05)

Values are Pearson's correlation coefficient (*P*‐value).

DDI = Defect Distribution Index; VDP = Ventilation Defect Percentage; FEV_1_ = Forced Expiratory Volume in 1 second; FVC = Forced Vital Capacity; BLEO = childhood‐cancer survivors who received bleomycin; BMT = bone‐marrow transplantation; BOS = bronchiolitis‐obliterans syndrome; BPD = bronchopulmonary dysplasia; CF = cystic fibrosis; FLD = Fibrotic lung diseases; LAM = lymphangioleiomyomatosis.

### Comparison of DDI and VDP Ratio Across Groups

**FIGURE 5:**
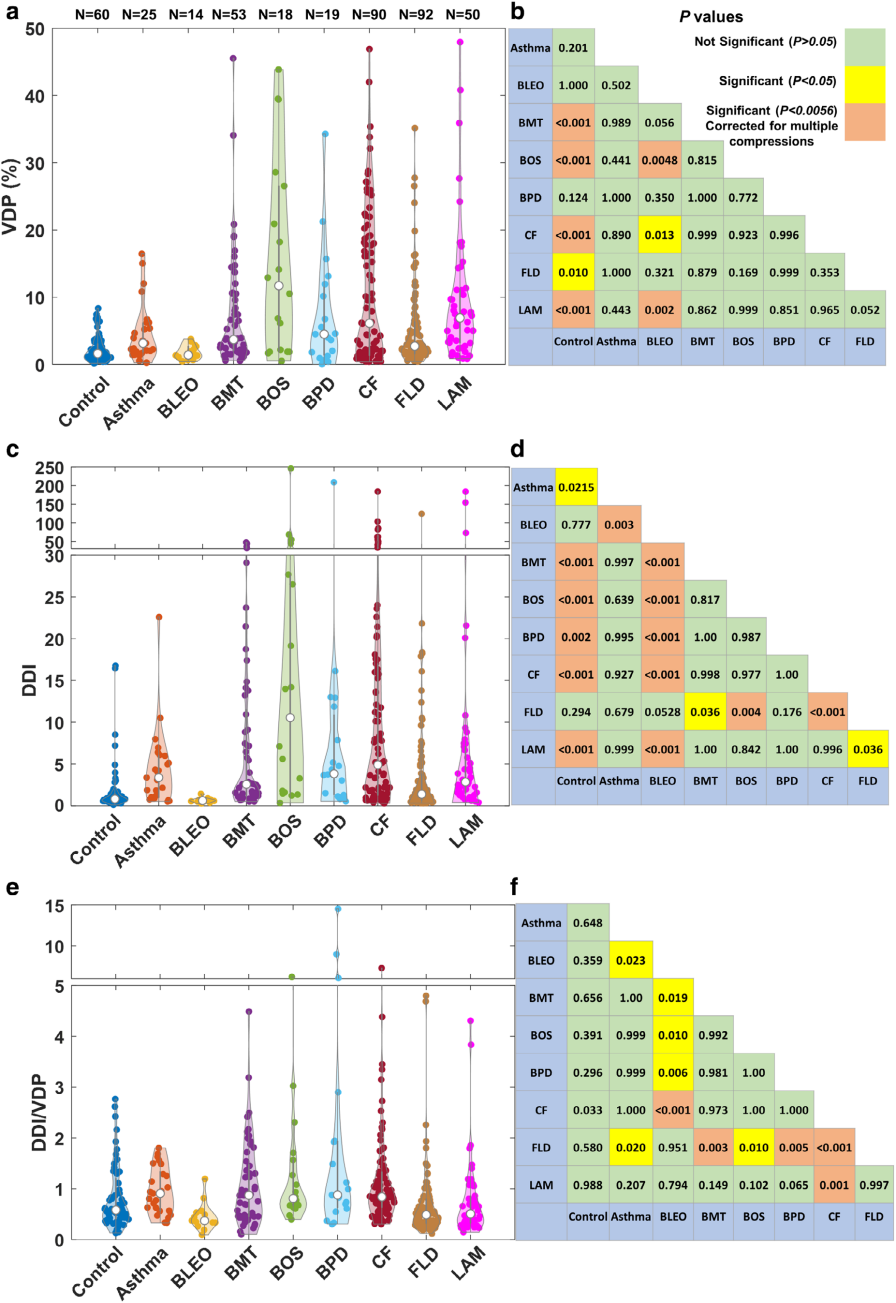
VDP, mean DDI maps and DDI/VDP across all groups. (**a**) Presents VDP and (**b**) depicts statistical comparisons of VDP between groups. (**c**) Presents mean DDI maps, while (**d**) depicts statistical comparisons of mean DDI between groups. Additionally, (**e**) and (**f**) display DDI/VDP and statistical comparisons of DDI/VDP between groups, respectively.

Figure 5a,b display violin plots of VDP values across all groups, accompanied by statistical comparisons between groups. VDP was significantly higher in the BMT, BOS, CF, and LAM groups than in the Control group. VDP was also significantly higher in the BOS and LAM groups than in the BLEO group. Mean DDI values across all groups are shown in Fig. 5c,d, accompanied by statistical comparisons between groups. Mean DDI was significantly higher in the BMT, BOS, BPD, CF, and LAM groups than in the Control and BLEO groups. Additionally, the Asthma group had a significantly higher mean DDI than the BLEO group. Furthermore, mean DDI in the FLD group was significantly lower than mean DDI in the BOS and CF groups. These findings highlight distinct variations in mean DDI across different patient groups. Additionally, mean DDI was significantly higher in axial slices compared to coronal slices (Fig. S7 in the Supplemental Material) in both the Control and the FLD groups. DDI/VDP was also significantly higher in axial slices compared to coronal slices in the FLD. In contrast, in the CF group, mean DDI was significantly higher in coronal slices compared to axial slices, while DDI/VDP did not differ significantly between coronal and axial slices (*P* = 0.53).

To facilitate DDI comparisons across all groups while adjusting for VDP differences, Fig. 5e,f shows violin plots of the DDI/VDP ratio, with statistical comparisons between the groups . DDI/VDP in the CF group was significantly higher compared to the BLEO and LAM groups. DDI/VDP in the FLD group was significantly lower than DDI/VDP in the BMT, BPD, and CF groups. Moreover, although not shown in the figure, the maximum values of DDI and DDI/VDP (Table 2) exhibited the same trend as the mean values, reinforcing the consistency of these patterns across the studied groups.

**FIGURE 6:**
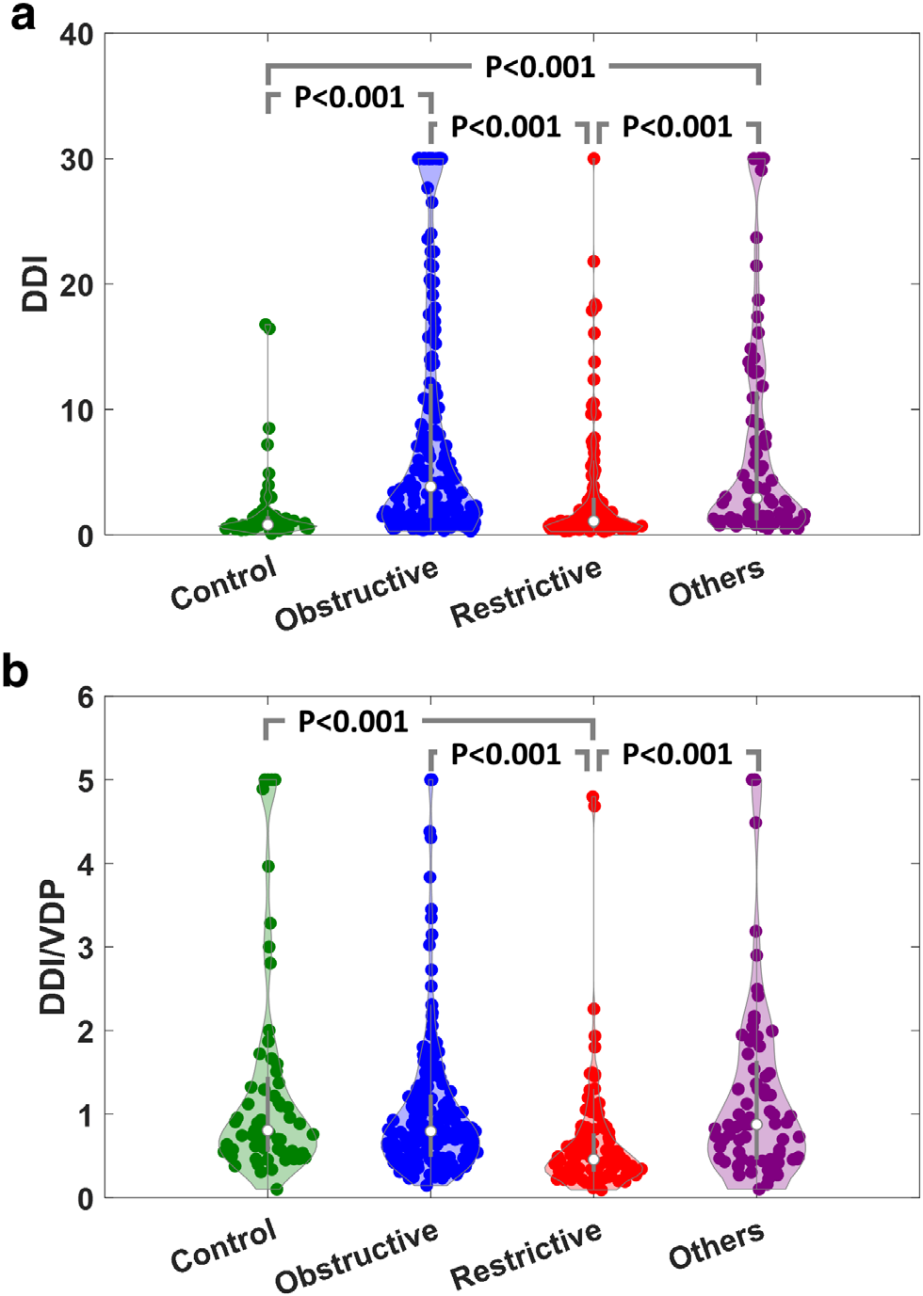
Comparisons of mean DDI and DDI/VDP across control and disease type groups. (**a**) Mean DDI is significantly higher in obstructive and other groups compared to controls and restrictive groups. (**b**) DDI/VDP is significantly lower in the restrictive group compared to controls, obstructive, and others. Note, outliers were adjusted to 30 in (a) and 5 in (b) to improve plot visualization only.

When categorizing diseases based on their primary respiratory disease pattern, mean DDI was significantly higher in the obstructive and others (BMT and BPD) groups compared to control and restrictive groups (Fig. 6a). Conversely, DDI/VDP was significantly lower in the restrictive group compared to controls, obstructive, and others groups (Fig. 6b).

## Discussion

VDP quantifies lung defects but does not fully capture the spatial and size distribution necessary to understand regional lung pathophysiology or the impact of airway size and generation on ventilation. A method to systematically assess the spatial distribution of defects provides additional insights into localized lung dysfunction, which is crucial for evaluating targeted therapies such as thermoplasty or endobronchial valves.^28^

A direct approach to assessing regional distribution of defects involves reporting VDP within each lung lobe. However, the spatial resolution of HP gas images is modest relative to modalities such as computed tomography (CT), so the fissures that demarcate lobe boundaries are not readily apparent. Attempts have made to address this limitation by dividing lungs into six regions based on lung geometry.^29^ Nonetheless, this method falls short of providing a comprehensive understanding of the spatial distribution of defects. Additionally, a previously described approach involves reporting the largest defect and the number of defects using HP gas images in patients with CF.^14^ However, this method still lacks details on the spatial distribution of defects across the entire lung volume.

In contrast, DDI,^15^ quantifies the spatial distribution of defects. The validity of this method has been confirmed through human visual scoring of defects seen on proton images in patients with CF.^15^ It was also applied in functional matrix‐pencil decomposition (MP) lung MRI.^30^ While 2D DDI maps exhibit sensitivity in quantifying the spatial distribution of defects on a slice level, they do not capture the intrinsically 3D nature of the defects. Following the distribution analyses of clustered emphysema‐like tissues on CT images,^31^ the approach reported in the current study incorporates expanding spheres in the analysis of ventilation defects, as opposed to expanding circles, to enable the quantification of DDI in three dimensions.

Moreover, various methods have been employed to quantify spatial clustering in medical imaging, including spatial statistics like the K‐function and nearest neighbor analysis, which assess clustering by comparing observed patterns to random distributions.^32^ Texture analysis techniques such as the Gray Level Co‐occurrence Matrix (GLCM) and Gray Level Run Length Matrix (GLRLM) also provide valuable insights into spatial patterns and clustering.^33^, ^34^ However, DDI offers a distinct advantage in lung imaging by specifically addressing the unique characteristics of ventilation defects. Unlike general spatial statistics or texture measures, DDI is tailored to quantify clustering directly within the context of lung ventilation data, enhancing the precision and relevance of clustering analysis for clinical interpretation.

The spatial distribution of lung defects across diseases may offer insights into the underlying pathophysiology and severity of each condition. A sparse distribution, characterized by a low DDI, may serve as an indicator of milder and/or early disease. This pattern suggests that defects are dispersed or less concentrated in specific areas of the lungs. In diseases exhibiting a sparse distribution, certain lung regions may maintain relatively normal ventilation, reflecting a better overall lung function. Conversely, a focal distribution, associated with a high DDI, signifies substantial localized impairment. A high DDI may imply uneven ventilation, with some areas experiencing significant ventilation defects while others remain relatively unaffected. In specific conditions, a focal defect distribution might also be linked to advanced disease stages (depending on the defect size), where specific regions are more severely affected. The distribution of lung defects, whether sparse or focal, may offer insights into the nature and severity of respiratory abnormalities across various lung diseases. Such insights may be important as clinical trial endpoints or secondary endpoints.

In our study, mean DDI was correlated with VDP in all but the BLEO group, and with PFTs in the CF, FLD and LAM groups. The mean DDI had significant correlation with VDP largely because DDI measures clustered defects, making it inherently related to VDP. This result agrees with Valk et al.^15^ However, DDI is sensitive to differences in ventilation distribution for lung images with similar VDPs indicating that, despite correlation, VDP and DDI quantify different aspects of the same images.

DDI did not correlated significantly with PFTs except in the CF, FLD, and LAM groups. This is not completely surprising because PFTs are insensitive to regional disease^35^, ^36^ and interrogate a different aspect of lung physiology (i.e., primarily upper airway function). In contrast, DDI assesses a distinct aspect of lung impairment, focusing on defect distribution. Consequently, DDI serves as a complementary parameter to existing lung function measures, and its inclusion alongside these parameters may provide additional insights into lung disease severity and progression.

While lung diseases differ etiologically and clinically, the significant differences observed in DDI/VDP across various disease groups shed light on distinct pathophysiology underlying each condition. The higher DDI/VDP in the CF group compared to the BLEO and LAM groups suggests a more localized and potentially clustered pattern of ventilation defects relative to lung volume. This finding likely reflects the multifaceted nature of CF lung pathology, which is characterized by chronic airway obstruction mucus plugging and airway remodeling.^37^ In contrast, the significantly lower DDI/VDP in the FLD group indicates a more widespread and heterogeneous distribution of ventilation defects compared to other groups. This may be attributed to specific etiological factors unique to fibrotic lung diseases, such as interstitial fibrosis and scarring, leading to more dispersed changes in lung architecture.^38^ Overall, these findings underscore the importance of considering both the extent and distribution of ventilation defects in understanding the pathophysiology of different lung diseases and tailoring treatment strategies accordingly.

The observed disparity in mean DDI and DDI/VDP between axial and coronal slices across Control, CF, and FLD groups highlights the complexity of lung ventilation dynamics and the influence of slice orientation on imaging outcomes. The lung structure is not perfectly symmetrical. The larger central airways and blood vessels are located more medially, with progressive branching toward the periphery. This can lead to inherent ventilation heterogeneity between the top and bottom of the lung.^39^ Axial slices transect the lung perpendicular to the long axis of the trachea, potentially capturing a greater proportion of poorly ventilated regions near the central airways. In contrast, coronal slices might sample a more balanced distribution of airways across the lung. Additionally, depending on the specific disease process, ventilation defects might be more concentrated in specific lung regions (eg, CF develop more severe lung disease in the upper lobes^40^). Axial slices could be more likely to intersect these areas, leading to a higher DDI compared to coronal slices that might average across both well and poorly ventilated zones.

Higher mean DDI was observed in the obstructive and other groups compared to controls and restrictive groups, suggesting distinct variations in the spatial clustering of defects among different patient groups. In particular, it indicates a higher degree of spatial clustering of ventilation defects in obstructive diseases. From a clinical and physiological perspective, this may indicate obstructed airways preclude ventilation into subsequent conducting airway generations and lead to larger ventilation defects. Conversely, restrictive diseases may primarily affect interstitial tissue and terminal airways, resulting in smaller, more dispersed defects. Note, this broader classification was chosen to simplify data presentation and provide a clearer framework for interpreting results relative to diseases not directly discussed in the manuscript.

Recent studies have highlighted the use of DDI as outcome measures following Highly Effective Modulator Therapy (HEMT) in patients with CF. For instance, the study by Streibel et al^41^ evaluated the impact of elexacaftor/tezacaftor/ivacaftor therapy on lung function and MRI‐derived parameters in CF patients using proton imaging. The results demonstrated significant improvements in DDI for both ventilation and perfusion, indicating a reduction in the heterogeneity of these defects. This finding suggests that DDI metrics can serve as sensitive indicators of regional lung function improvement post‐HEMT. Additionally, the study emphasized that while traditional measures like FEV_1_ improved, the DDI metrics provided additional insight into the spatial distribution of ventilation and perfusion abnormalities, highlighting their potential as complementary outcome markers in monitoring CFTR‐modulator therapy efficacy in clinical practice.

### Limitations

We acknowledge the limitation of small sample sizes, particularly in the BLEO (N = 14). The reduced number of participants may affect the statistical power and generalizability of our findings, making them more variable and less representative of the broader population. As such, these results should be interpreted with some caution, and additional studies with larger groups are needed. Additionally, ^129^Xe ventilation images often exhibit anisotropic voxel size, with variations in slice thickness and gaps between slices. Although the 3D DDI approach employed in this study compensated for this by interpolating the slices, true 3D isotropic^42^, ^43^ images would be preferable for accurate quantification of defect distribution.

A second limitation arises from the binary nature of the input (Eq. A2 in Appendix A in the Supplemental Material) for DDI calculation. This eliminates information about image texture and signal intensity distribution that could serve as markers for impaired ventilation. Future research could mitigate these limitations by integrating both non‐binary defect maps and image features into the analysis pipeline. Finally, lung function varies with age in both health and disease and therapy status. However, age dependence was not accounted for in this study, which involves participants ranging in age from 6 to 80 years, potentially confounding DDI results. Further, our data collection spanned a long period, during which new therapies—especially HEMT for CF—came into routine clinical use. As such, time‐varying clinical care over may have altered lung function, impacting the quantitative DDI results.

## Conclusions

In contrast to the qualitative descriptions typically offered by radiologists, DDI facilitates a quantitative analysis of defect distributions. Further, 3D DDI provides a means to assess whole‐lung function and provides quantitative information beyond conventional PFTS and VDP. The DDI variations between obstructive and restrictive lung diseases reflect differences in the spatial distribution and concentration of ventilation defects, potentially providing information about the underlying lung biology and pathophysiology associated with these distinct respiratory conditions.

## Supporting information


**Data S1:** Supporting Information.
