# Supplementary material for: Quantifying Spatial Distribution of Ventilation Defects in Multiple Pulmonary Diseases With Hyperpolarized 129Xenon MRI
Source: J Magn Reson Imaging. 2024 Oct 22;61(4):1860–73. doi: 10.1002/jmri.29627 (PMC11896935; doi:10.1002/jmri.29627)
Supplement: Supplementary file 1 — Data S1: Supporting Information. [file JMRI-61-1860-s001.docx]

**SUPPLEMENT**

**Quantifying Spatial Distribution of Ventilation Defects in Multiple Pulmonary Diseases with Hyperpolarized ^129^Xenon MRI**

**Appendix A: Theory of Defect Distribution Index**

Defect distribution index (DDI) is a technique introduced by Valk *et. al* (1) to quantify defect distribution in 2D proton imaging by placing a circle around each defected voxel and gradually increasing its radius until the fraction of defected voxels within the radius falls below 50% of the total voxels inside. Building on this effort, we adapted this approach for 3-dimessions by using spheres instead of circles. To account for anisotropic voxels size, we interpolated (nearest neighbor) the slices according to the slice thickness between slices until isotropic voxels size is achieved.

Each voxel within the segmented lung mask, $seg\left( x_{i} \right)$, can be represented as

$seg\left( x_{i} \right)= \left\{ \begin{aligned} 0:background \\ >0:lung \end{aligned} \right.$*,* (A1)

and the defected voxels, $dm\left( x_{i} \right)$, are represented as

$dm\left( x_{i} \right)= \left\{ \begin{aligned} 1:no defects \\ 2:defects \end{aligned} \right.$*.* (A2)

To enable comparisons across diverse scales (e.g., pediatric vs. adult lung size), lung volume, $V_{lung}$ is calculated as mean number of lung voxels across the $S$ slices of the lung:

$V_{lung}= \frac{\sum_{1}^{S} \sum seg\left( x_{i} \right)}{S}.$ (A3)

To determine the defect ratio within a sphere centered on the target defect voxel with radius r, $DR\left( x_{i},r \right)$, we calculate it by dividing the number of defected voxels $D\left( x_{i},r \right)$ by the total number of voxels $V\left( x_{i},r \right)$ whose centers are contained within the sphere of radius r:

$D\left( x_{i},r \right)= \left\{ x_{j}|dm\left( x_{j} \right)=2{\wedge x}_{j}\in A\left( x_{i},r \right) \right\}$, (A4)

$V\left( x_{i},r \right)= \left\{ x_{j}|\left\| x_{i}-x_{j} \right\|_{2} \leq r \right\}$, (A5)

$DR\left( x_{i},r \right):=\frac{\left| D\left( x_{i},r \right) \right|}{\left| V\left( x_{i},r \right) \right|}$*.* (A6)

From Eq. A6, a cluster score as a function of $r$ can be calculated as:

$C\left( x_{i},r \right):=100\cdot\frac{DR\left( x_{i},r \right)-T}{\left( 1-T \right)}$. (A7)

The parameter $T$ determines the minimum defect ratio needed for a voxel group to qualify as a cluster. $T$ was set to 0.5 to strike a balance in defining clusters based on this criterion, following the rational provided by Valk *et. al* (1)*.*

The normalized volume $N\left( x_{i},r \right)$ as a function of $r$ can be calculated as:

$N\left( x_{i},r \right):=\frac{\left| V\left( x_{i},r \right) \right|}{V_{lung}}$. (A8)

Finally, a stepwise integration of the discrete values of cluster score $C\left( x_{i},r \right)$ as function of $N\left( x_{i},r \right)$ is performed as the value of $r$ increases. This increment continues as long as the value of $C\left( x_{i},r \right)$ remains positive (>50% of voxels in sphere are defects). Given this series of increasing radii $r_{0},r_{1},\ldots,r_{n}$ with distinct corresponding $V\left( x_{i},r \right)$ values, where $r_{n}$ is the largest radius with a positive value of $C\left( x_{i},r \right)$, the DDI is calculated as follows:

$DDI\left( x_{i} \right):=\sum_{k=1}^{n} C\left( x_{i},r_{k} \right)\cdot\left( N\left( x_{i},r_{k} \right)- N\left( x_{i},r_{k-1} \right) \right).$ (A9)

Mean of DDI maps can then be computed for each slice and across the whole lungs.


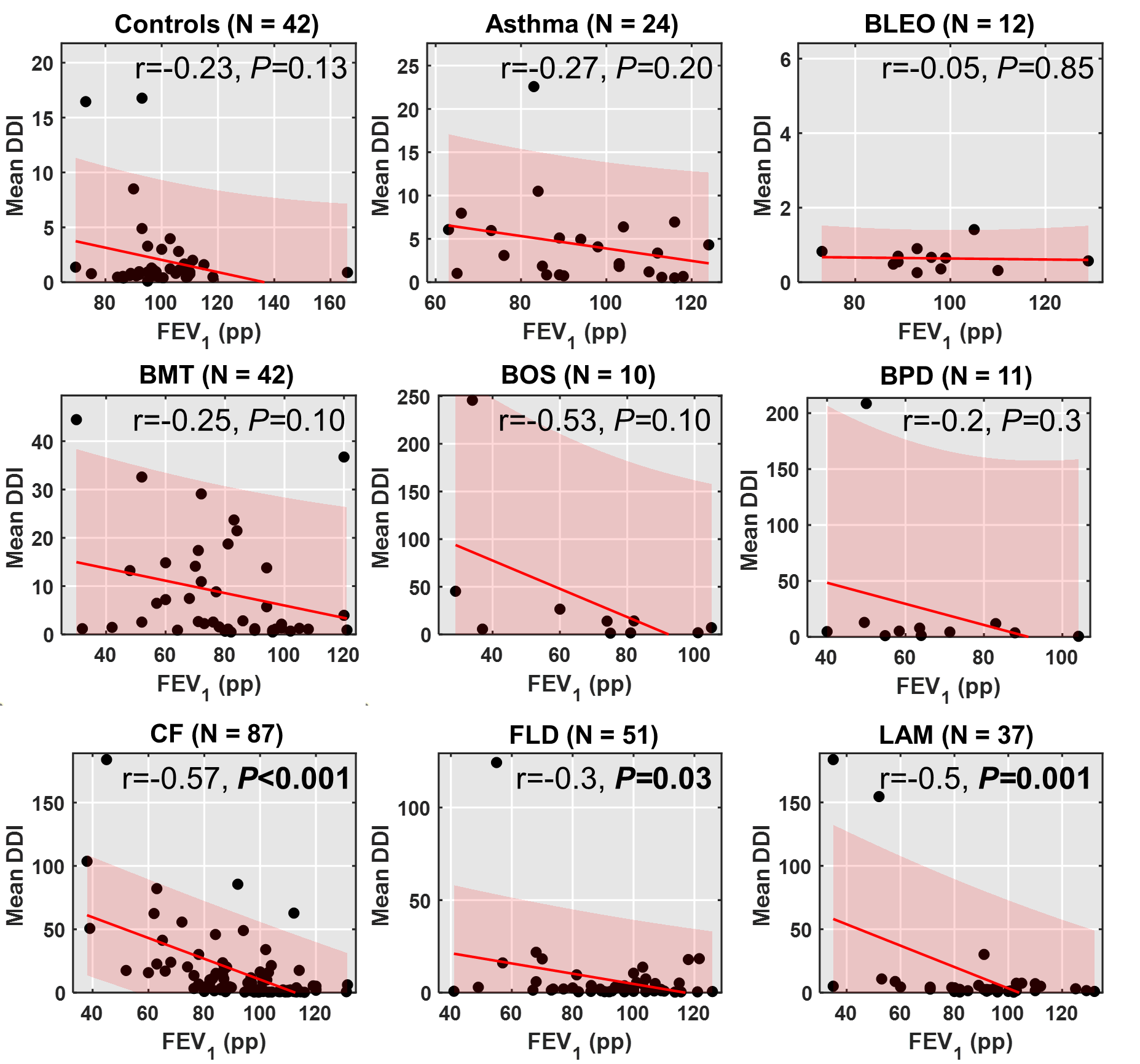


Figure S1. Scatter plots depicting the correlations between DDI and FEV_1_ across all groups. In most groups, DDI did not correlate significantly with FEV_1_ (r ≤ -0.05, *P* ≥ 0.10), except for CF, FLD and LAM, where the correlation reaches significance (*P* ≤ 0.03). The 95% confidence interval is shaded in red, and the least squares fit is represented by the red line.


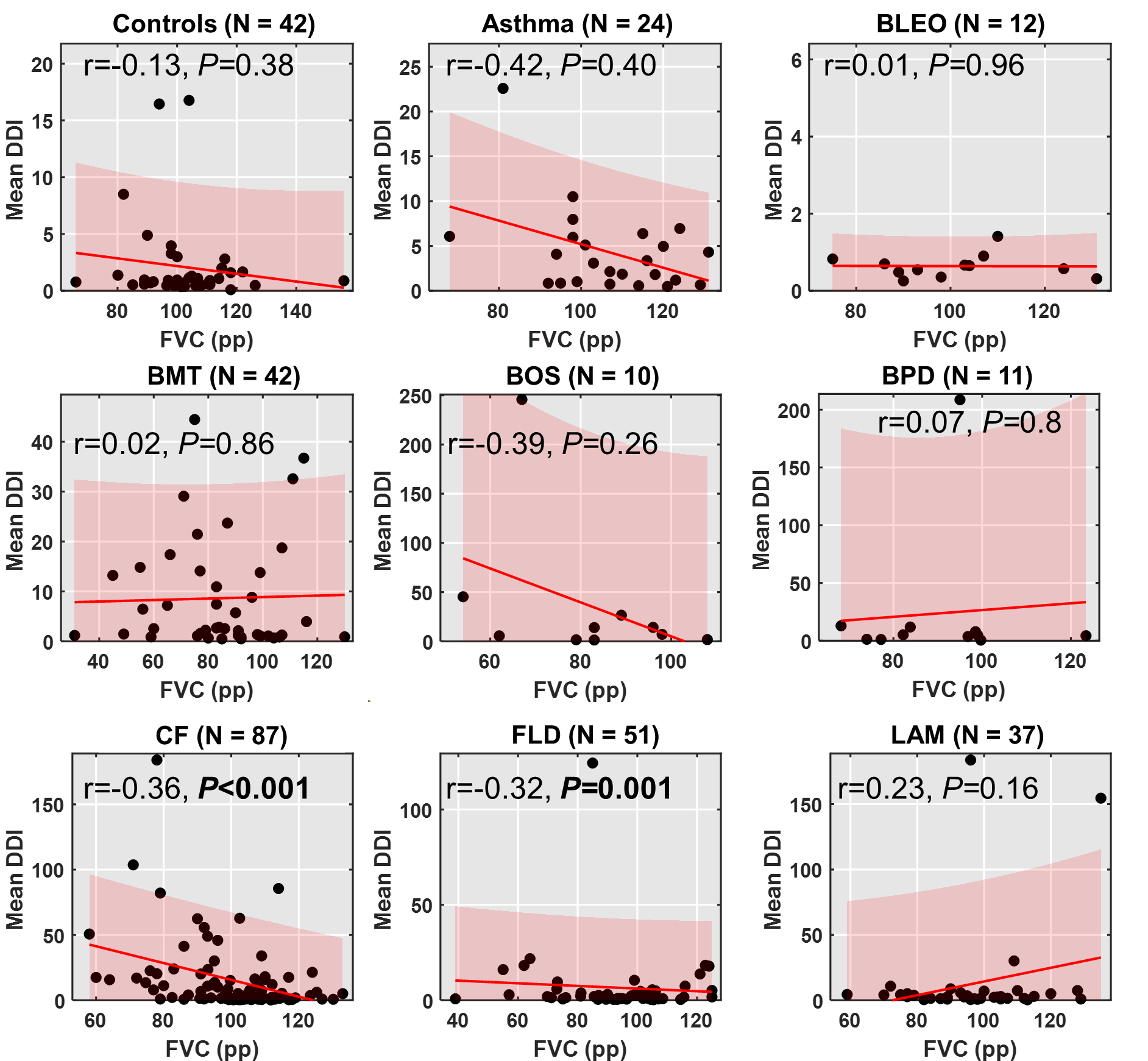


Figure S2. Scatter plots depicting the correlations between DDI and FVC across all groups. In most groups, DDI did not correlate significantly with FVC (r ≤ 0.23, *P* ≥ 0.16), except for CF, and FLD, where the correlation is significant (*P* ≤ 0.001). The 95% confidence interval is shaded in red, and the least squares fit is represented by the red line.


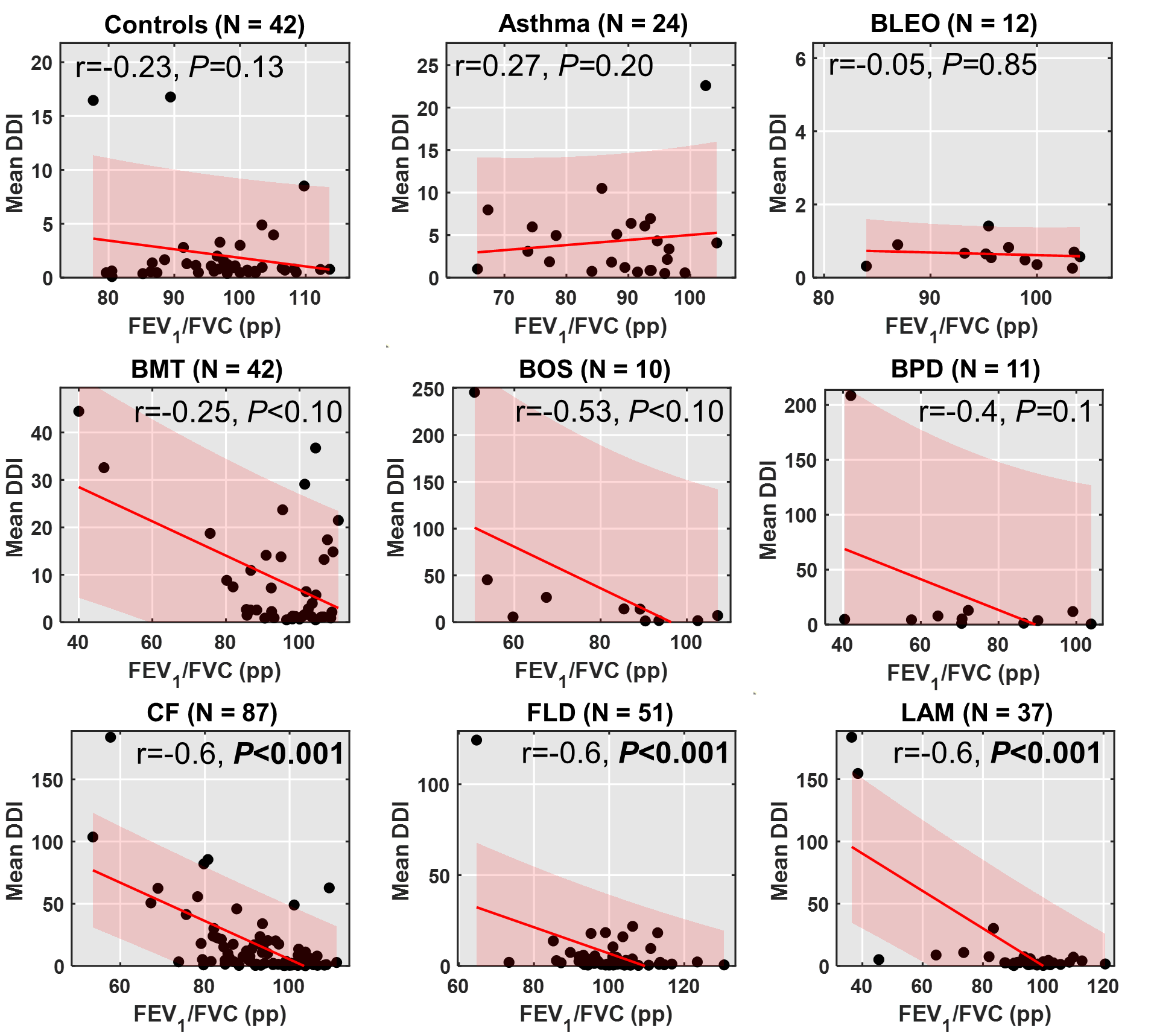


Figure S3. Scatter plots depicting the correlations between DDI and FEV_1_/FVC across all groups. In most of groups, DDI did not correlate significantly with FEV_1_/FVC (r ≤ 0.27, *P* ≥ 0.10), except for CF, FLD and LAM, where the correlation is significant (*P* ≤ 0.001). The 95% confidence interval is shaded in red, and the least squares fit is represented by the red line.


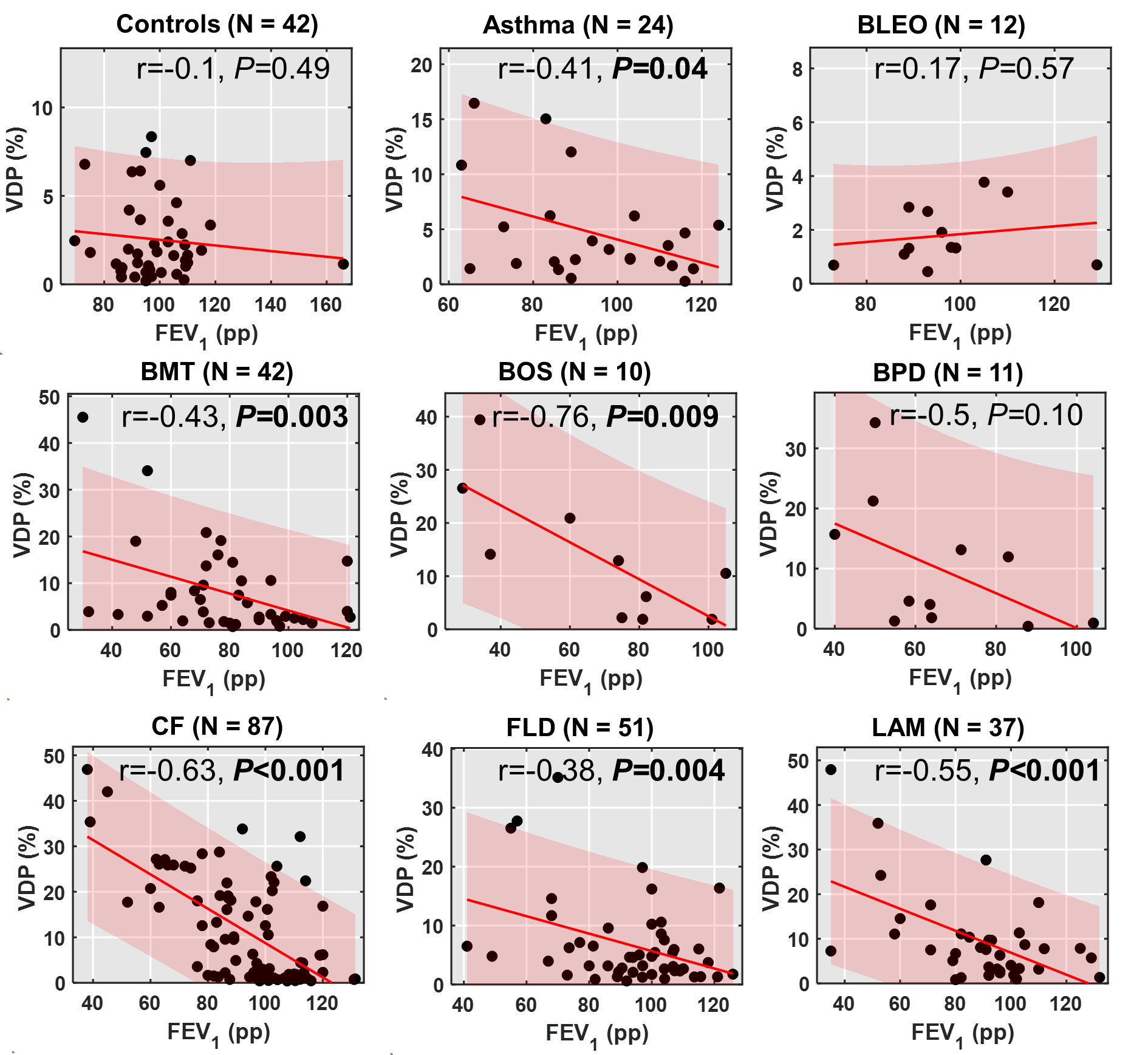


Figure S4. Scatter plots depicting the correlations between VDP and FEV_1_ across all groups. In the majority of groups, VDP correlated significantly with FEV_1_, except for control, BLEO and BPD. The 95% confidence interval is shaded in red, and the least squares fit is represented by the red line.


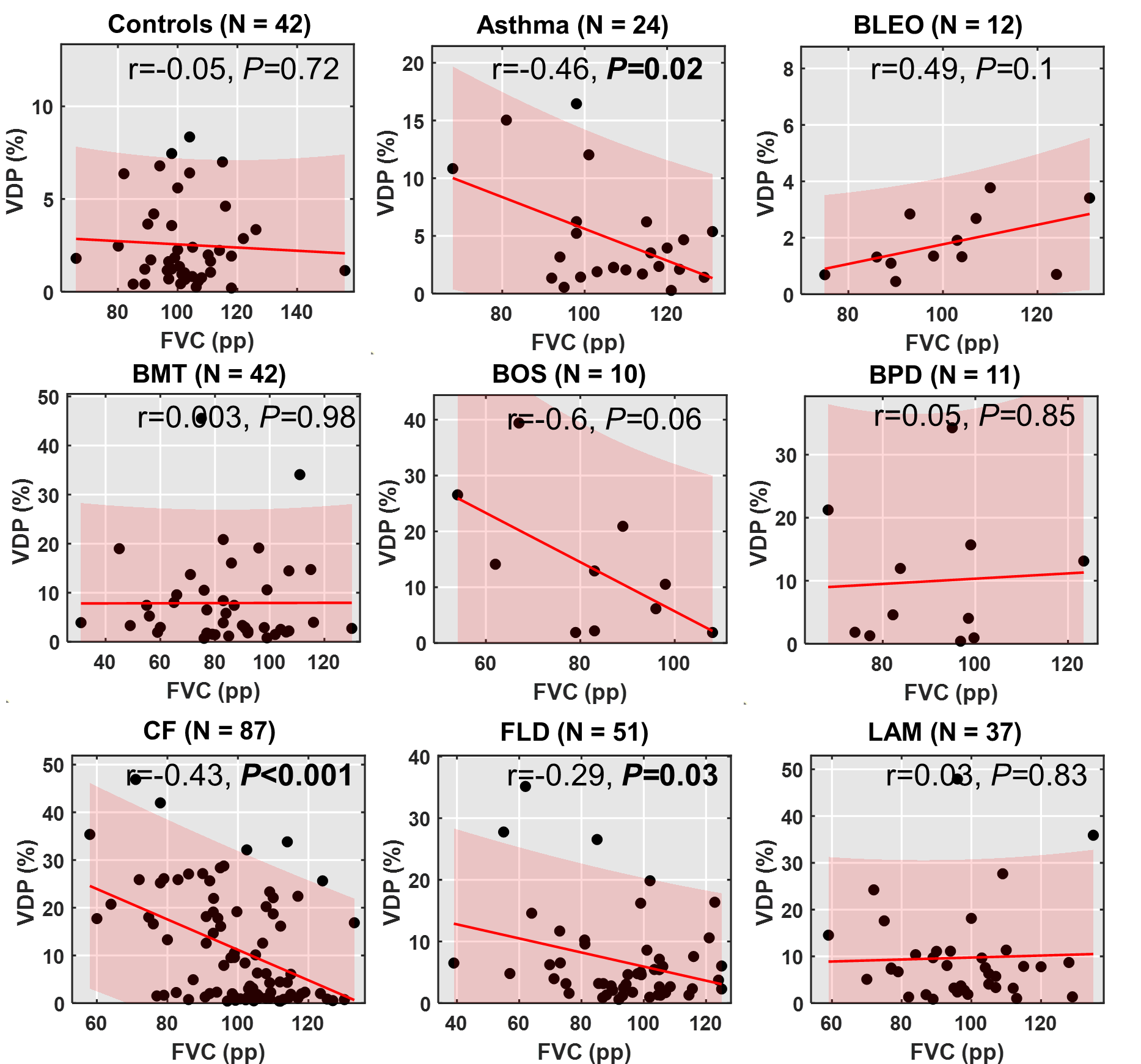


Figure S5. Scatter plots depicting the correlations between VDP and FVC across all groups. VDP correlated significantly with FVC in Asthma, CF and FLD only. The 95% confidence interval is shaded in red, and the least squares fit is represented by the red line.


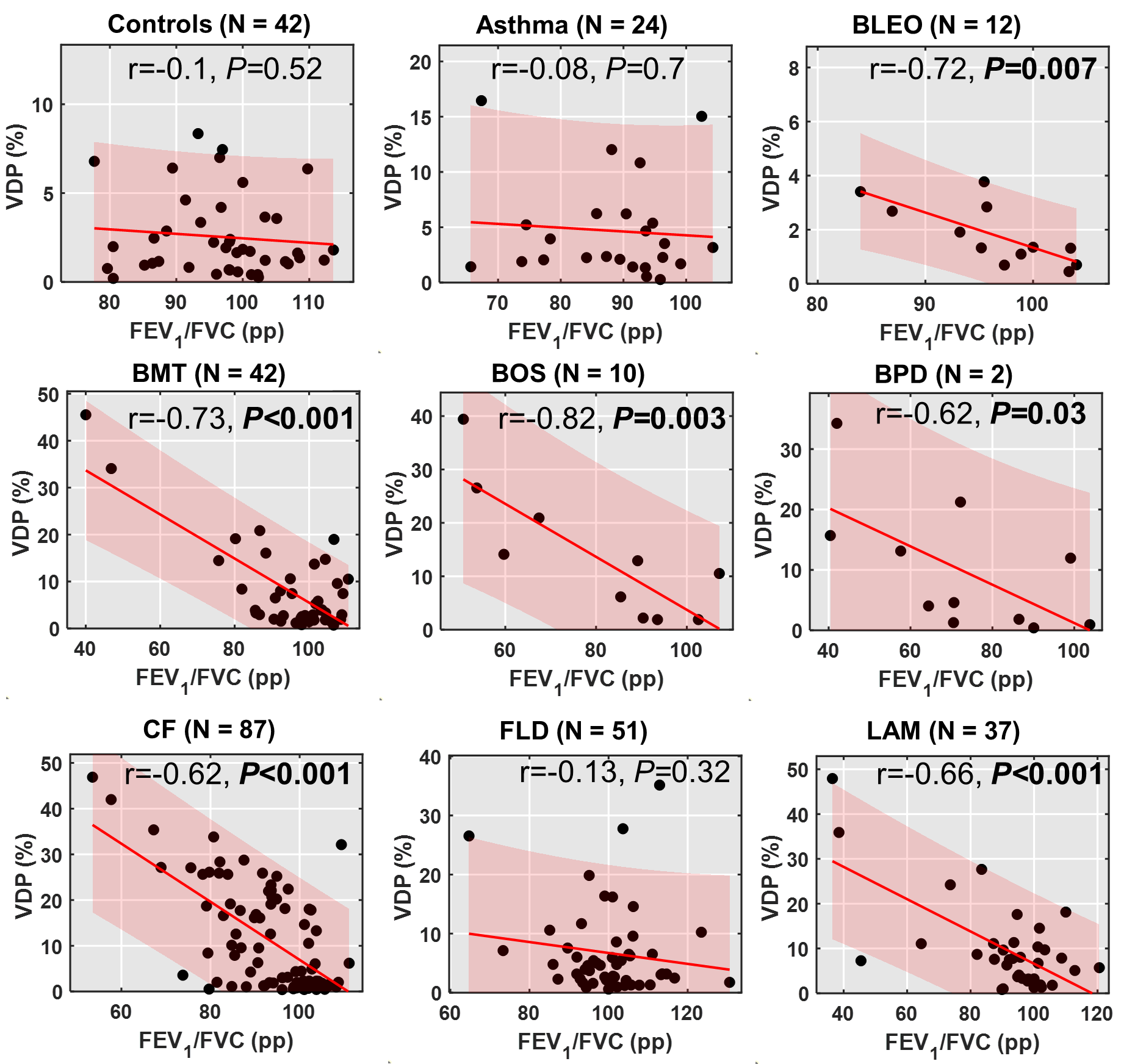


Figure S6. Scatter plots depicting the correlations between VDP and FEV_1_/FVC across all groups. In most groups, VDP correlated significantly with FEV_1_/FVC, except for control, Asthma and FLD. The 95% confidence interval is shaded in red, and the least squares fit is represented by the red line.


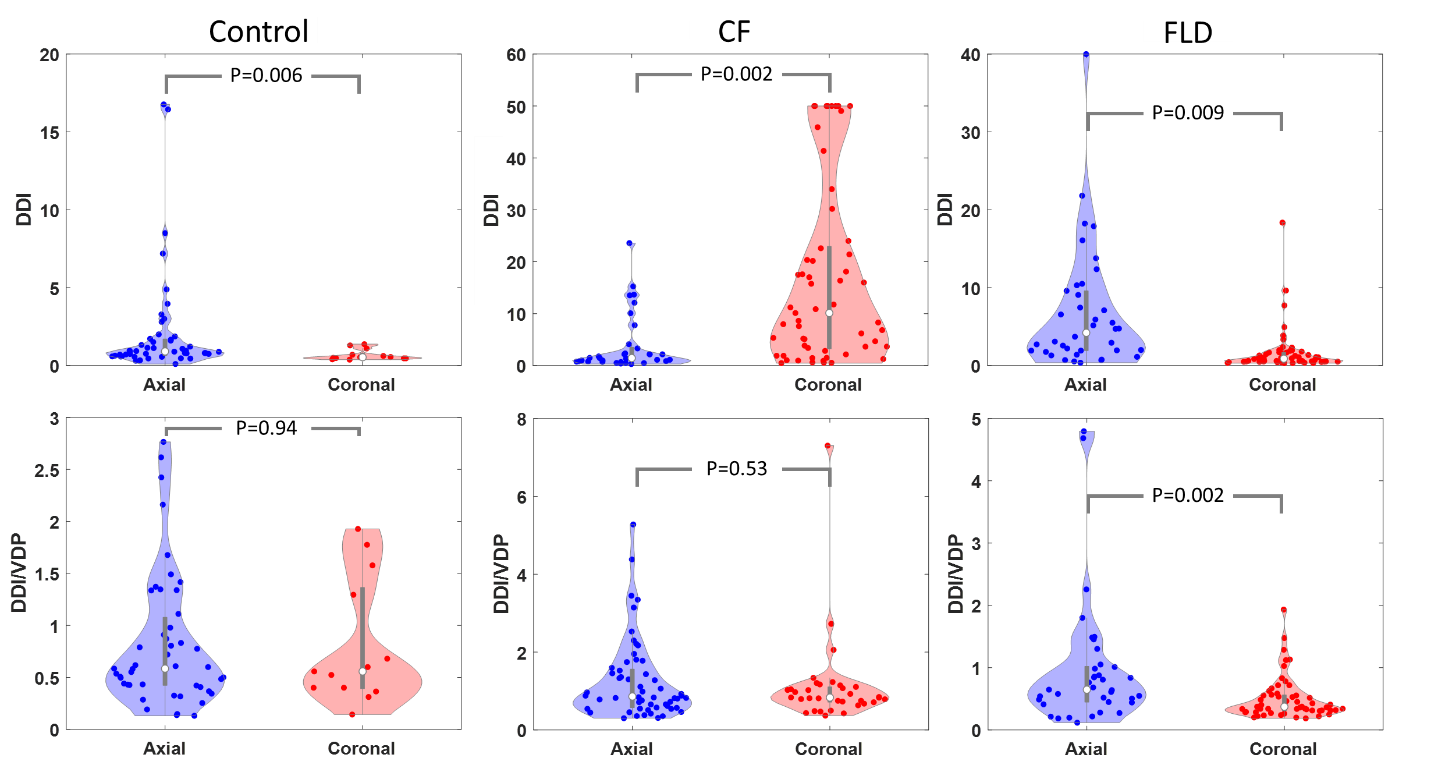


Figure S7. DDI and DDI/VDP comparison between slice orientations for control, CF and FLD. Both DDI and DDI/VDP were significantly higher in axial slices for control and FLD groups. However, the CF group displayed an opposite trend, with a significantly higher mean DDI in coronal slices compared to axial slices (*P* = 0.002). DDI/VDP in the CF group did not show a significant difference between slice orientations (*P* = 0.53).

**References**

1. Valk A, Willers C, Shahim K, Pusterla O, Bauman G, Sandkühler R, Bieri O, Wyler F, Latzin P. Defect distribution index: A novel metric for functional lung MRI in cystic fibrosis. Magnetic resonance in medicine 2021;86(6):3224-3235.
